# Half-Barrels Derived from a (β/α)_8_ Barrel β-Glycosidase Undergo an Activation Process

**DOI:** 10.1371/journal.pone.0139673

**Published:** 2015-10-02

**Authors:** Daniela Beton, Sandro R. Marana

**Affiliations:** Departamento de Bioquímica, Instituto de Química, Universidade de São Paulo, São Paulo, Brazil; Russian Academy of Sciences, Institute for Biological Instrumentation, RUSSIAN FEDERATION

## Abstract

The evolution of (β/α)_8_ barrel proteins is currently thought to have involved the fusion of two (β/α)_4_ half-barrels, thereby conferring stability on the protein structure. After the formation of a whole (β/α)_8_ barrel, this structure could evolve and diverge to form fully active enzymes. Interestingly, we show here that isolated (β/α)_4_ half-barrels derived from the N- and C-terminal domains of the β-glucosidase Sfβgly (Sfβgly-N: residues 1 to 265; Sfβgly-C: residues 266 to 509) undergo an activation process, which renders them catalytically active. The rate constants of the activation process were calculated to be 0.029 and 0.032 h^-1^ for Sfβgly-N and Sfβgly-C, respectively. Moreover, the Sfβgly-N and Sfβgly-C activation processes were simultaneous with modifications in their initial structure, which reduced the exposure of their tryptophan residues. Importantly, this activation was also coincident with an increase in the sizes of Sfβgly-N and Sfβgly-C particles. These novel observations suggest that the change in catalytic activity associated with the transition from a half to whole (β/α)_8_ barrel might also have driven such an evolutionary process.

## Introduction

The TIM (or (β/α)_8_ barrel) is the most common fold in enzymes, and enzymes containing this fold function as hydrolases, oxidoreductases, lyases, transferases or isomerases [1,2]. Of those enzymes, the glycoside hydrolases represent the largest group of enzymes that adopt a (β/α)_8_ barrel fold [1]. Because of its wide distribution, the evolution of this fold has been intensely discussed in the literature [1,3,4]. It has been proposed that the (β/α)_8_ barrel evolved from the duplication and fusion of a half-barrel (β/α)_4_ unit or alternatively resulted from two sequential duplication and fusion events that started from a quarter barrel (β/α)_2_ unit [2,3,5,6,]. Most studies on the*in vitro* reconstruction of those events have focused on the structure of the initial half-barrels, and again in stability is assumed to have driven (β/α)_8_ barrel evolution [3,2]. Indeed, the fusion of two (β/α)_4_ half-barrels could have contributed to the formation of the active site at the C-terminal top of the barrel, which then could have diverged into different enzymes. Indeed, the (β/α)_4_ half-barrels that have been characterized have not exhibited enzymatic activity when isolated, but when two complementary (β/α)_4_ half-barrels were co-expressed or co-refolded, an active whole (β/α)_8_ barrel was regenerated [6–8]. Nevertheless, as (β/α)_8_ barrels are mainly enzymes, characterization of the enzymatic activity in the transition from pieces to whole barrels is a relevant question that needs to be addressed.

Herein, we worked with (β/α)_4_ half-barrels derived from the N- and C-terminal halves of the β-glucosidase Sfβgly (AF052729) [9], a member of Glycoside Hydrolase family 1. We show that those (β/α)_4_ half-barrels undergo an activation process, which renders them catalytically active. Moreover, such activation processes occur simultaneously with modifications in the initial structure of the (β/α)_4_ half-barrels and are coincident with an increase in size. These observations suggest that in addition to the gain in structural stability, the increase in catalytic activity might also have driven the evolution of (β/α)_8_ barrels.

## Material and Methods

### Assembly of the Expression Vectors for Sfβgly-N and Sfβgly-C

The cDNAs encoding Sfβgly-N (residues 1 to 265 of Sfβgly) and Sfβgly-C (residues 266 to 509 of Sfβgly) were amplified using a PCR Master Mix kit (Fermentas Life Science; Vilnius, Lithuania) and a pAE vector containing an insert for native Sfβgly as template. The reaction cycles (30) were 30 s at 94°C, 60 s at 60°C and 90 s at 72°C. The pair of primers 5ʹ-CCGCTCGAGCAGCAGCGCCGCTTC-3-ʹ and 5ʹ-CCCAAGCTTTCATTCAGCAGCCATTTCATCC-3ʹ was used for Sfβgly-N, whereas the primers 5ʹ-CCGCTCGAGCTTAGGAGACAGGGTGAGTGG-3ʹ and 5ʹ-CCCAAGCTTTCAATGTCCCTCATCTATAGTCATG-3ʹ were used for Sfβgly-C. The amplified products were analyzed by electrophoresis in 1% agarose gels and extracted using a PCR clean-up kit (Promega; Fitchburg, USA). Purified products were digested with the enzymes *Xho*I and *Hind*III, for which sites were present in the primers (underlined and double underlined segments, respectively). Next, digested products were cloned into a pAE vector [10], which had been previously digested with the same restriction enzymes. The complete ligation reaction was used to transform XL1 Blue competent cells, which were cultivated on Luria broth agar plates containing 50 μg/mL ampicillin. Transformed colonies were cultivated in 5 mL of Luria broth containing 50 μg/mL ampicillin for 16 h. Next, they were used for plasmid extraction using a DNA purification kit (Promega Fitchburg, USA). Plasmids were sequenced using Big Dye termination mix (Applied Biosystems; Waltham, USA).

### Expression and Purification of Sfβgly-N and Sfβgly-C in ArticExpress (DE3)

A colony of ArticExpress(DE3) freshly transformed with either the vector pAE-Sfβgly-N or pAE-Sfβgly-C was transferred to 10 mL of 2xTY medium (1.6% tryptone, 1% yeast extract and 8.5 mM NaCl) containing 50 μg/mL carbenicillin and 10 μg/mL gentamicin and cultivated at 37°C for 20 h at 200 rpm. Next, the culture medium was diluted (1:20) with 100 mL of fresh 2xTY medium without antibiotics and incubated again under the same conditions. When this culture reached an OD_600nm_ = 0.4, IPTG was added to a final concentration of 1 mM, and the temperature was reduced to 12°C. After 24 h of induction under these conditions, bacteria were harvested by centrifugation (4000×*g* for 20 min at 4°C) and stored at –80°C. For purification of recombinant proteins, pellets of induced bacteria were resuspended in 1 mL of lysis buffer (10 mM sodium phosphate buffer, pH 7.0 containing 100 mM NaCl, 10 mM imidazole and 1 mM PMSF). This suspension was subjected to ultrasound (3 pulses of 15 s) in a Branson sonifier 450 with a microtip (set at output 3). The suspension was cooled on ice for 1 min between each pulse. Finally, the suspension was centrifuged (10,000×*g* for 30 min at 4°C). The supernatant (~1 mL) was collected, and 0.2 mL of Ni-NTA agarose (Qiagen; Venlo, Netherlands), which had been previously equilibrated in 10 mM sodium phosphate buffer, pH 7.0, was added. Proteins that were not retained in the resin were removed by washing in 0.75 mL of 10 mM sodium phosphate buffer, pH 7.0, and centrifugation (16,000×*g* for 1 min). This washing step was repeated 5 times, and the final pellet, the resin containing the recombinant proteins that were specifically bound, was stored on ice for the next step. Finally, recombinant proteins were eluted in 0.15 mL of 10 mM sodium phosphate buffer, pH 7.0, containing 300 mM imidazole. The buffer of the supernatant containing eluted proteins was exchanged to 50 mM sodium citrate-phosphate buffer, pH 6.0, using a HiTrap desalting column (GE HealthCare; Little Chalfont, UK).

### SDS–PAGE and Immunoblotting

The protein electrophoresis in denaturing conditions were as described elsewhere [11]. For visualization, proteins were stained using 0.2% (m/v) Coomassie Blue R prepared in 50% methanol and 2% acetic acid (v/v). For immuno-detection, proteins were transferred to nitrocellulose membranes [12]. Transfer was confirmed by staining the membranes with 0.1% Ponceau-S (m/v) prepared in 10% acetic acid (v/v). Membranes were destained using water and blocked with 5% skimmed milk prepared in 10 mM Tris-HCl, pH 7.4, containing 150 mM NaCl (TBS). For immune detection of the recombinant proteins, the membranes were incubated for 16 h at 4°C with anti-His HRP antibody (Qiagen; hearafter termed anti-His_6_) prepared in TBS containing 5% skimmed milk. Next, free antibodies were washed out by incubation (6 steps of 10 min) in TBS containing 0.1% Tween 20 (m/v). Recombinant proteins specifically recognized by the anti-His_6_ antibody were stained using an Opti-4CN kit (BioRad; Hercules, CA, USA).

### β-Glucosidase Assay

The enzymatic activity of β-glucosidase was detected using as a substrate 1 mM methylumbelliferyl β-glucoside prepared in 100 mM citrate-sodium phosphate buffer, pH 6.2. The reaction mixture containing substrate and enzyme was incubated at 30°C for defined periods of time. Reactions were stopped with 200 mM glycine-NH_4_OH, pH 10.5, and the product methylumbelliferone was detected by fluorescence (excitation, 360 nm; emission, 450 nm).

### Determination of Rate Constants for the Activation Process

The activation processes of Sfβgly-N and Sfβgly-C were analyzed according to the hysteresis model [13], which results in Eq 1 for determining the activation rate constant.

ln(v2−vt)=ln(v2−v1)⋅−kt (equation 1)

The rates of substrate hydrolysis at different times (*v*_t_) were determined by calculating the slope of [P] *versus* time curve. The value *v*_t_ is proportional to the concentration of active enzyme at time t. The value *v*_2_ is proportional to the total concentration of enzyme, which was calculated based on the slope at the highest experimental time (48 h). *v*_1_ is the initial activity of the inactive enzyme, which was assumed to be 0 because the [P] *versus* time converges to zero at time zero. Thus, *v*_t_, *v*_2_ and *v*_1_, which were calculated from the experimental [P] *versus* time curve, were linearized using a plot of ln (*v*_2_-*v*_t_) *versus* t, for which the slope corresponds to the activation rate constants (*k*).

### Determination of Circular Dichroism Spectra

Protein samples were prepared in 10 mM potassium phosphate buffer, pH 6.9, and transferred to 0.5 mm quartz cells. CD spectra from 310 to 190 nm were collected at 25°C in a spectropolarimeter Jasco J-720. The scan speed was set to 50 nm/min, bandwidth to 1.0 and scanning mode to continuous. Residual molar ellipticity was calculated as previously described [14]. CD spectra were deconvoluted using K2d software [14].

### Determination of Fluorescence Spectra for Proteins

Protein samples were incubated in a quartz cell (10 mm) at 30°C in a F4500 espectrofluorometer (Hitachi; Tokyo, Japan). Samples were excited at 295 nm, and emission spectra were detected from 305 to 400 nm. The scan speed was set to 1.2 nm/min, the integration time to 1 s and the slit opening to 5.0 nm. For fluorescence quenching experiments, spectra were determined in the presence of acrylamide (up to 0.47 M) prepared in 50 mM citrate-sodium phosphate buffer, pH 6.0. The maximal fluorescence values were recorded at the same wavelength in the absence (F_0_) or presence (F) of acrylamide. The ratios F_0_/F at each acrylamide concentration [Q] were plotted as previously described [15] for the determination of the Stern–Volmer constant (*K*_sv_). Quenching experiments were performed with protein samples previously incubated at 30°C in 50 mM citrate-sodium phosphate buffer, pH 6.0, for 0 and 48 h.

### Dynamic Light Scattering

Protein samples prepared in 50 mM citrate-sodium phosphate buffer, pH 6.0, were passed through 0.22 μm filters and transferred to disposable plastic cuvettes (50 μL). Samples were previously incubated at 30°C in 50 mM citrate-sodium phosphate buffer, pH 6.0, for 0, 24 and 48 h. Data were collected at 30°C using a Zetasizer Nano ZS (Malvern; Worcestershire, UK).

## Results and Discussion

**Fig 1:**
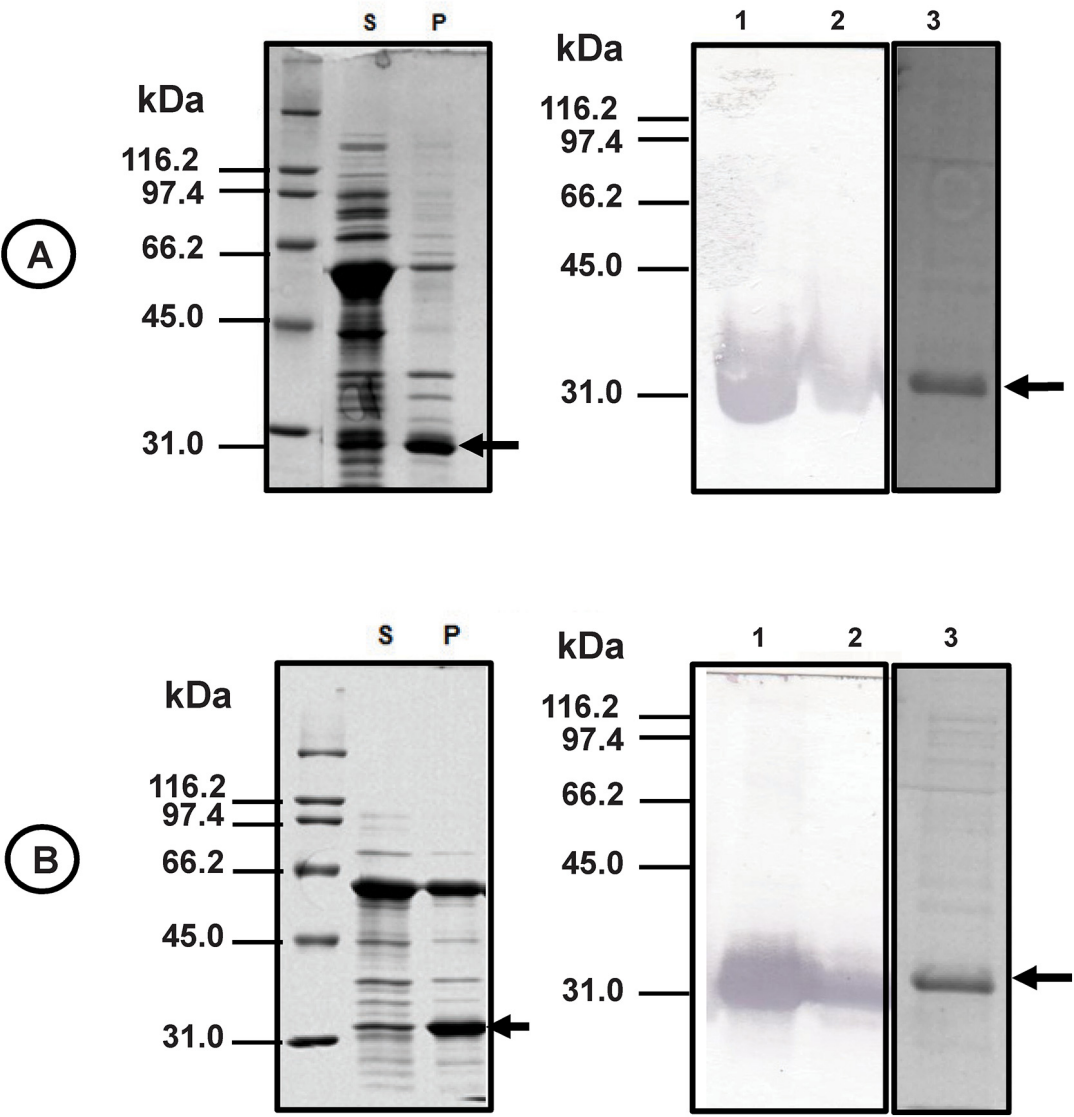
Expression and purification of Sfβgly-N and Sfβgly-C. Proteins from the soluble (lane S) and insoluble (lane P) fractions of bacteria (ArticExpress (DE3)) induced to produce Sfβgly-N **(A)** and Sfβgly-C **(B)** were analyzed by SDS–PAGE (12% polyacrylamide; Comassie Blue R250 staining), transferred to a nitrocellulose membrane and immuno-detected using anti-His_6_ antibody (lanes 1 and 2, respectively). Sfβgly-N and Sfβgly-C were purified from the respective soluble fractions using Ni-NTA agarose and analyzed by SDS–PAGE (lane 3; arrow; Comassie Blue R250 staining).

The cDNA coding the N- and C-terminal halves (residues 1 to 265 and 266 to 509) of the GH1 β-glucosidase Sfβgly [9] (AF052729) was amplified by PCR and cloned into the expression vector pAE. Considering that Sfβgly folds as a (β/α)_8_ barrel, these halves are hereafter termed Sfβgly-N and Sfβgly-C, which are predicted to correspond to (β/α)_4_ half-barrels. Thus, each of these half-barrels, as well as native Sfβgly, were produced as recombinant proteins in ArticExpress(DE3) bacteria at 12°C for 24 h. Next, they were successfully purified using Ni-NTA agarose resin. The homogeneity and identity of Sfβgly-N and Sfβgly-C were confirmed by SDS–PAGE and western blotting using anti-His_6_ antibody (Fig 1). Approximately 200 μg of both soluble Sfβgly-N and Sfβgly-C was obtained from 3 L induced bacterial media. However, the yields were variable, and freshly transformed bacteria had to be used for each induction. Notably, Sfβgly-N and Sfβgly-C were also expressed in BL21-Gold (DE3) bacteria at 20, 25 and 37°C, but no soluble recombinant protein was obtained from those induced bacteria.

**Fig 2:**
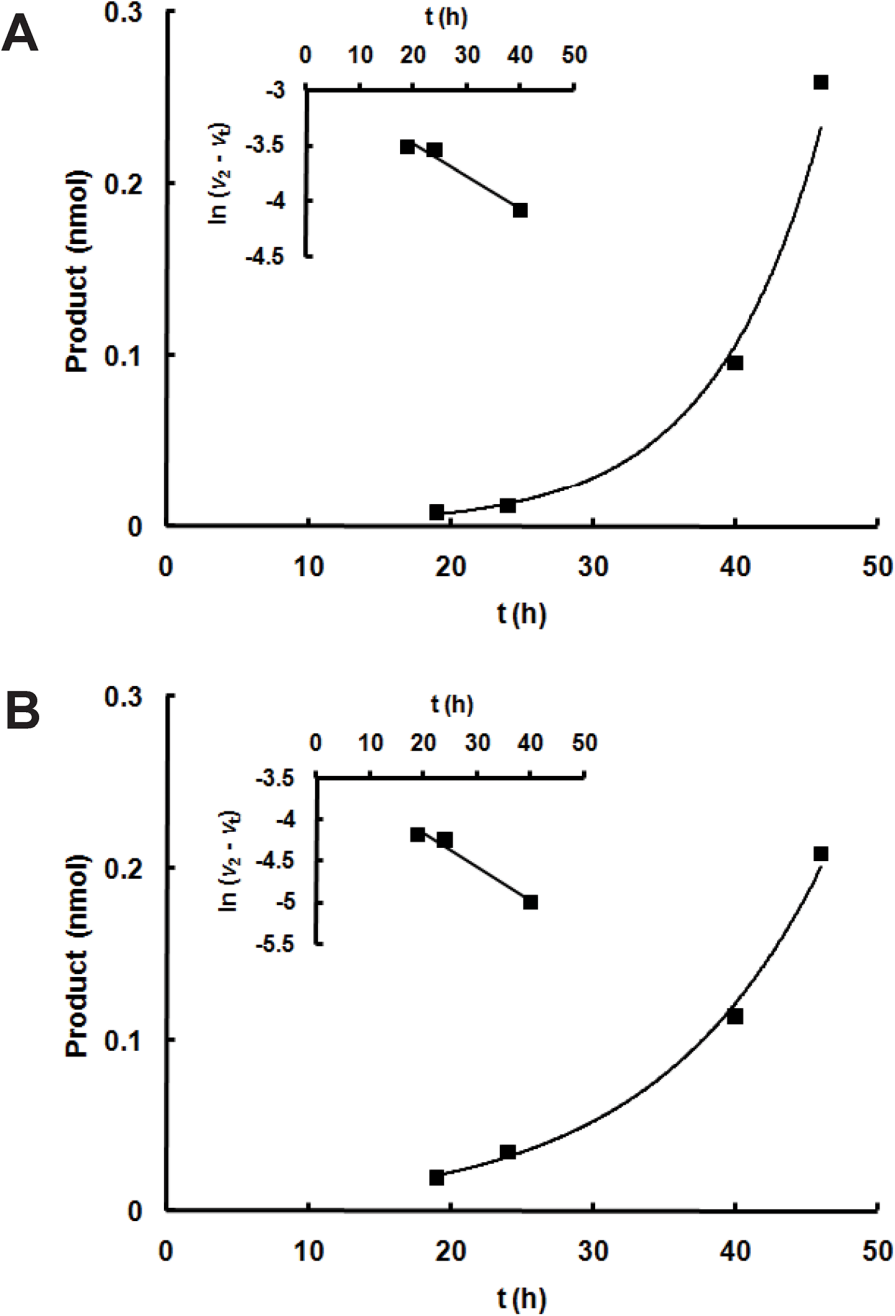
Enzyme activity assay of Sfβgly-N and Sfβgly-C showing an activation process. Purified samples of Sfβgly-N **(A)** and Sfβgly-C **(B)** in 100 mM citrate-phosphate buffer, pH 6.0, were incubated with substrate 1 mM methylumbelliferyl β-glucoside prepared in 100 mM citrate-phosphate buffer, pH 6.0, for 16, 22, 40 and 48 h. The fluorescence of the product methylumbelliferone (excitation, 360 nm; emission, 450 nm) was determined after the addition of 100 mM glycine-NaOH, pH 10.5. **Inserts:** rates were analyzed according the hysteresis model [10]. The reaction rate at each time (*v*_t_) was calculated based on the slope at that specific time. The slope at 48 h was taken as the final rate (*v*_2_).

Sfβgly-N and Sfβgly-C were subjected to enzymatic activity assays using methylumbelliferyl β-glucoside as a substrate. Remarkably, both half-barrels exhibited hydrolytic activity toward that substrate, which increased with time and yielded exponential curves (Fig 2). These findings suggest that the concentration of catalytically active Sfβgly-N and Sfβgly-C increased with assay time, indicating that these half-barrels underwent an activation process. Native Sfβgly did not exhibit such behavior in similar activity assays actually generating linear curves as expected. Moreover, control assays prepared with substrate alone, in which the half-barrel samples were replaced by buffer containing 300 mM imidazole,did not show β-glucosidase activity. Finally, mock “Sfβgly-N and Sfβgly-C samples” produced from extracts of ArticExpress (DE3) bacteria transformed with an “empty” pAE vector were used in the β-glucosidase assays, and these assays showed no product formation. Accordingly, mock “Sfβgly-N and Sfβgly-C samples” produced from ArticExpress (DE3) bacteria expressing an inactive mutant Sfβgly showed no product formation.

The Sfβgly-N and Sfβgly-C activation process was analyzed based on the hysteresis model [13], which describes enzyme activation by chemical or physical modifications that occur on the same time scale as the catalytic activity. Thus, simultaneously with product formation, the concentration of active enzyme increases and results in exponential curves of product *versus* time. Hence, increases in rate, which are proportional to the concentration of active enzyme at each reaction time, were graphically analyzed (Fig 2, inserts), resulting in the rate constants 0.029 and 0.032 h^-1^ for the activation processes of Sfβgly-N and Sfβgly-C, respectively (Table 1). These data indicate that those half-barrels needed 35 and 33 h, respectively, to be converted into an active form.

**Table 1:** Kinetic parameters for the activation of Sfβgly-N and Sfβgly-C.

	Sfβgly-N	Sfβgly-C
*k* (h^-1^)	0.029 ± 0.006	0.032 ± 0.007

Rate constants (*k*) for the activation process were calculated based on seven independent assays.

This observation is in agreement with previous studies showing that half-barrels showed no enzymatic activity just after purification because indeed Sfβgly-N and Sfβgly-C slow activation process was only detected after a long incubation time using a sensitive activity assay based on the formation of a fluorescent product.

It is unclear how isolated Sfβgly-N and Sfβgly-C can catalyze a hydrolytic reaction, as native GH1 β-glucosidases depend on two glutamic acid residues, catalytic acid/base and nucleophile, for that activity [16]. However, the observation of an activation process suggests that a modified form of these half-barrels is active.

**Fig 3:**
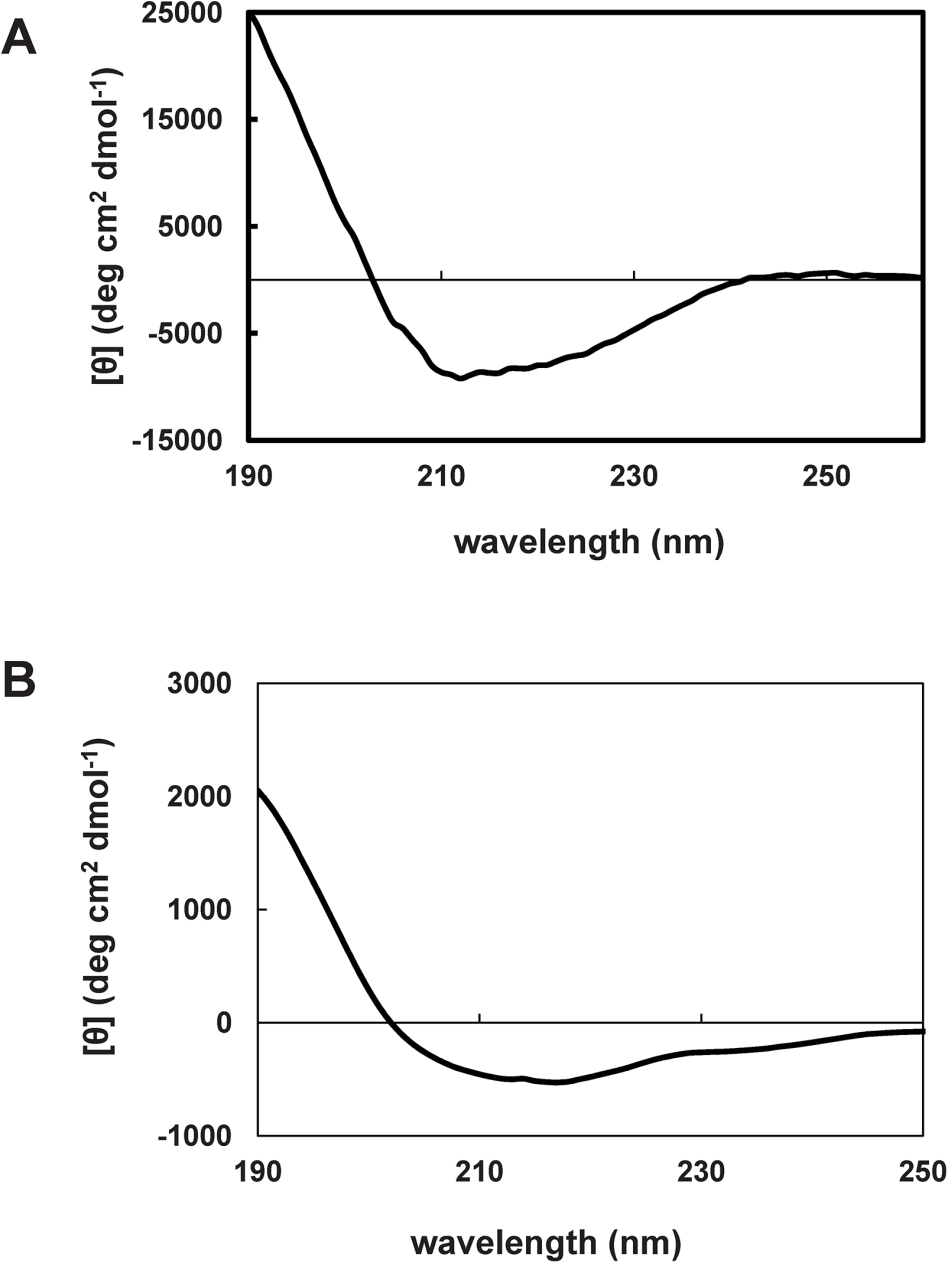
Circular dichroism spectra for Sfβgly-N and Sfβgly-C. Purified samples of 0.22 mg/mL Sfβgly-N **(A)** and 0.25 mg/mL Sfβgly-C **(B)** prepared in 10 mM potassium phosphate buffer, pH 6.9, were used for spectrum determination at 25°C (n = 8 reads).

To understand the putative modifications that Sfβgly-N and Sfβgly-C are subjected to during the activation process, we physically characterized these half-barrels. Initially, circular dichroism spectra indicated that both half-barrels had a secondary structure (Fig 3). Sfβgly-N is mainly composed of α-helices (64%) and unstructured regions (33%), whereas β-strands are a minor component (3%). Conversely, Sfβgly-C is mainly composed of unstructured regions (49%) and β-strands (47%), and α-helices are a minor component (4%).

**Fig 4:**
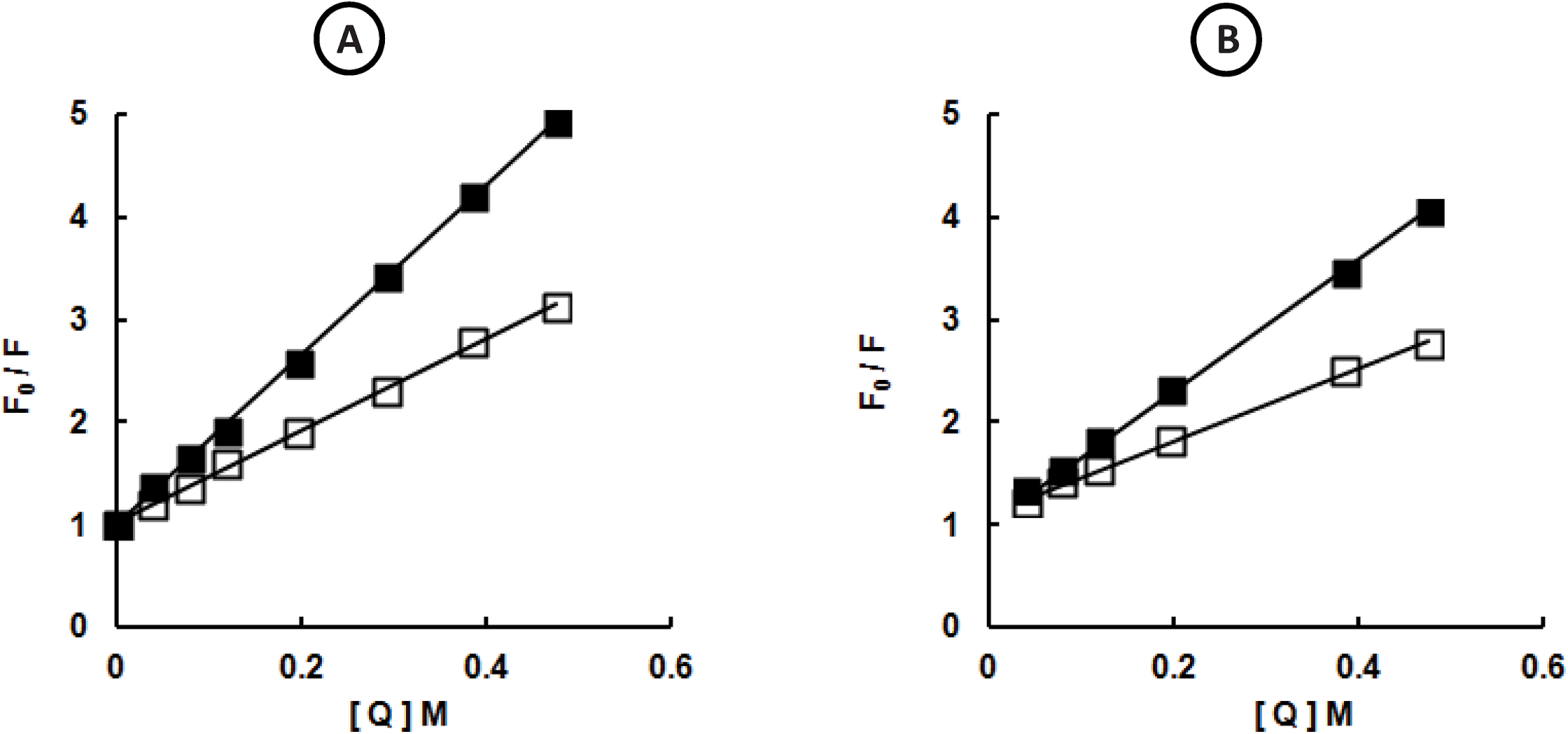
Acrylamide quenching of Sfβgly-N and Sfβgly-C fluorescence. Purified samples of Sfβgly-N **(A)** and Sfβgly-C **(B)** were used for the determination of fluorescence spectra (excitation, 295 nm; emission, 305 to 450 nm) at different concentrations of acrylamide ([Q], 0 to 0.47 M). The maximum emission was determined at the same wavelength in the absence (F_0_) and presence (F) of acrylamide. Spectra were determined after incubation for 0 (■) and 48 h (□) in 50 mM citrate-sodium phosphate buffer, pH 6.0.

Next, the modification of the Sfβgly-N and Sfβgly-C structure along with the activation process was verified using tryptophan fluorescence quenching experiments (Fig 4). The Stern-Volmer constants (*K*_sv_) for acrylamide quenching of Sfβgly-N intrinsic fluorescence decreased from 5.9 to 4.5 after 48 h of incubation in 50 mM citrate-sodium phosphate buffer, pH 6.0. Similarly, for Sfβgly-C, the *K*_sv_ was reduced from 6.5 to 3.8 after 48 h. Thus, for both half-barrels, 48 h of incubation in 50 mM citrate-sodium phosphate buffer, pH 6.0, which is sufficient for complete Sfβgly-N and Sfβgly-C activation, reduced the access of acrylamide to tryptophan residues [15], suggesting that the activation process involves structure modifications that reduce the exposure of Sfβgly-N and Sfβgly-C tryptophan residues. The *K*_sv_ for native Sfβgly remained unchanged (7.3 *versus* 7.5) after a 48 h incubation.

Finally, physical modifications occurring throughout the activation process for Sfβgly-N and Sfβgly-C were also probed by dynamic light scattering. We initially observed that 100% of the Sfβgly-N particles in the sample exhibited a hydrodynamic radius of 30.8 nm. After incubation for 24 h in 50 mM citrate-sodium phosphate buffer, pH 6, 97% of them remained unchanged (21 nm), whereas 3% increased to 68.7 nm (Table 2). Finally after 48 h, 100% of Sfβgly-N particles in the sample had changed to 53.7 nm. A similar result was obtained for Sfβgly-C. Initially, 100% of particles in the sample had a radius of 37 nm, and after 24 h, they remained unaltered (41 nm), whereas after 48 h, 100% of them exhibited a radius of 75.5 nm (Table 2). The polydispersivity of the samples, which was lower than 25%, indicates that Sfβgly-N and Sfβgly-C were homogeneous throughout the incubation period. Interestingly, samples of Sfβgly-N and Sfβgly-C that were incubated at 4°C for 48 h did not show any modification in hydrodynamic radius.

**Table 2:** Characterization of Sfβgly-N and Sfβgly-Cby dynamic light scattering.

	Incubation time (h)	Hydrodynamic radius(nm)	Polydispersivity (%)
	0 h	31	25
Sfβgly-N	24 h	68.7 (3%) and 21.5 (97%)	18
	48 h	53.7	19.8
	0 h	37	24
Sfβgly-C	24 h	41.8	30
	48 h	75.5	24

Therefore, simultaneously with the enzymatic activation process (Fig 2), Sfβgly-N and Sfβgly-C appear to increase in radius (Table 2), which could indicate an oligomerization process. We noted that the initial hydrodynamic radius (31 and 37 nm) was not compatible with the expected value (~9 nm) for monomeric Sfβgly-N and Sfβgly-C particles. Perhaps the difference results from the partially unstructured nature of Sfβgly-N and Sfβgly-C, as revealed by the circular dichroism spectra (Fig 3). Alternatively, Sfβgly-N and Sfβgly-C could be present as oligomers at the initial time. Nevertheless, although the real “initial aggregation state” of these particles is not known, it is clear that the initial radius of the Sfβgly-N and Sfβgly-C particles had increased after 48 h.

## Conclusions

In brief, enzyme activity assays showed that the concentration of active Sfβgly-N and Sfβgly-C increased with time, indicating that these half-barrels underwent an activation process. Physical characterization of Sfβgly-N and Sfβgly-C suggests that they can undergo oligomerization, which involves modification of their structure and culminates in active forms of these half-barrels. These observations add interesting data to the discussion of (β/α)_8_ barrel evolution, as it is currently proposed that the fusion of two (β/α)_4_ half-barrels confers stability on the protein structure because of the removal of hydrophobic surfaces from the aqueous solvent to the protein core [3, 17]. In addition the data presented here suggest that the increased catalytic activity in the transition from half to whole (β/α)_8_ barrels might also have driven that evolutionary process.
